# Increased cytochrome C threonine 50 phosphorylation in aging heart as a novel defensive signaling against hypoxia/reoxygenation induced apoptosis

**DOI:** 10.18632/aging.204159

**Published:** 2022-07-25

**Authors:** Fanqi Li, Haoxuan Sun, Xiaolong Lin, Qiuyu Li, Donghui Zhao, Zichao Cheng, Jinghua Liu, Qian Fan

**Affiliations:** 1 Department of Cardiology, Beijing An Zhen Hospital, Capital Medical University, and Beijing Institute of Heart, Lung, and Blood Vessel Disease, Beijing, China

**Keywords:** phosphorylation, apoptosis, aging, hypoxic/reoxygenated injury

## Abstract

Previous studies have shown that aging promotes myocardial apoptosis. However, the detailed mechanisms remain unclear. Our recent studies revealed that aging not only activates apoptosis, but also activates some anti-apoptotic factors. By quantitative phosphoproteomics, here we demonstrated that aging increases cytochrome c (Cytc) phosphorylation at threonine 50 (T50), a post-translational modification with unknown functional impact. With point mutation and lentivirus transfection, cardiomyocytes were divided into four groups: empty vector group, WT (wild type), T50E (as a phosphomimic variant), and T50A (non-phosphorylatable). TUNEL staining and flow cytometry were used to determine the apoptosis ratio in different groups after hypoxic/reoxygenated (H/R) treatment. The results showed that T50-phosphorylated Cytc suppressed myocardial apoptosis induced by H/R. Furthermore, Western Blot and ELISA measurements revealed that Cytc T50 phosphorylation inhibited caspase-9 and caspase-3 activity without altering caspase-8, BCL-2, BCL-XL, and Bax expression. In our study, we demonstrated that aging increases phosphorylation Cytc at T50 and this aging-increasing phosphorylation site can suppress H/R-induced apoptosis.

## INTRODUCTION

Acute myocardial infarction is one of the leading causes of death worldwide. Thanks to the rapid development of interventional technology, the number of people dying from acute myocardial infarction has decreased significantly. On the contrary, more and more people are suffering from post-ischemic heart failure [1]. Heart failure is the end-stage for all heart diseases, which seriously affects the quality life of patients and causes a substantial social and medical burden. As such, heart failure has become an urgent unmet medical need [2].

Substantial evidence indicates that apoptosis is the leading cause of ischemia-induced heart failure [3]. Our previous study demonstrated that aging increases myocardial ischemia/reperfusion-induced apoptosis in humans and rats [4]. Others have also reported that aging significantly promotes apoptosis [5, 6]. However, to date, the precise mechanisms linking aging to myocardial apoptosis remain unknown. Our previous work demonstrated that aging augmented reactive oxygen and reactive nitrogen species levels in ischemic/reperfused hearts [7]. Liu et al. reported that increased Omi/HtrA2 mRNA and protein expression in the aging rat myocardium aggravates ischemia/reperfusion injury by promoting myocardial apoptosis [8]. However, the above mechanism cannot fully explain the apoptosis-regulated effects of aging. The specific regulation mechanism of aging on myocardial apoptosis has become a hot topic in aging and cardiovascular disease research.

More interestingly, our previous work demonstrates that aging not only stimulates pro-apoptotic pathways in cardiomyocytes, but also activates some anti-apoptotic factors [4, 9, 10]. These results indicate that the aging regulation of apoptosis is complex. Integrative approaches, i.e., inhibiting pro-apoptotic pathways in a combination of further promoting anti-apoptotic pathways, may be required to achieve optimal protection.

In the present study, we performed proteomics on myocardial tissue obtained from young and old mice. Compared with the young group, the expression of 88 proteins was up-regulated, and 80 were down-regulated in old mice. At a post-translational modification level, phosphorylation levels were up-regulated at 445 sites and down-regulated at 1526 sites in the old group. Phosphorylation level changes were widespread and stable in each sample. More importantly, these changes occurred in many essential apoptotic regulatory proteins, including MAPK14, Akt1, mTORC2, and p21. Upon further analysis of these results, changes in Cytc phosphorylation at T50 caught our attention as Cytc phosphorylation is involved in mitochondrial function [11]. Surprisingly, this site has not been functionally annotated in aging-related pathology.

We found a new aging-increasing phosphorylation site by proteomics, which may be associated with aging-related myocardial apoptosis regulation.

## RESULTS

### Aging increases Cytc T50 phosphorylation

**Figure 1:**
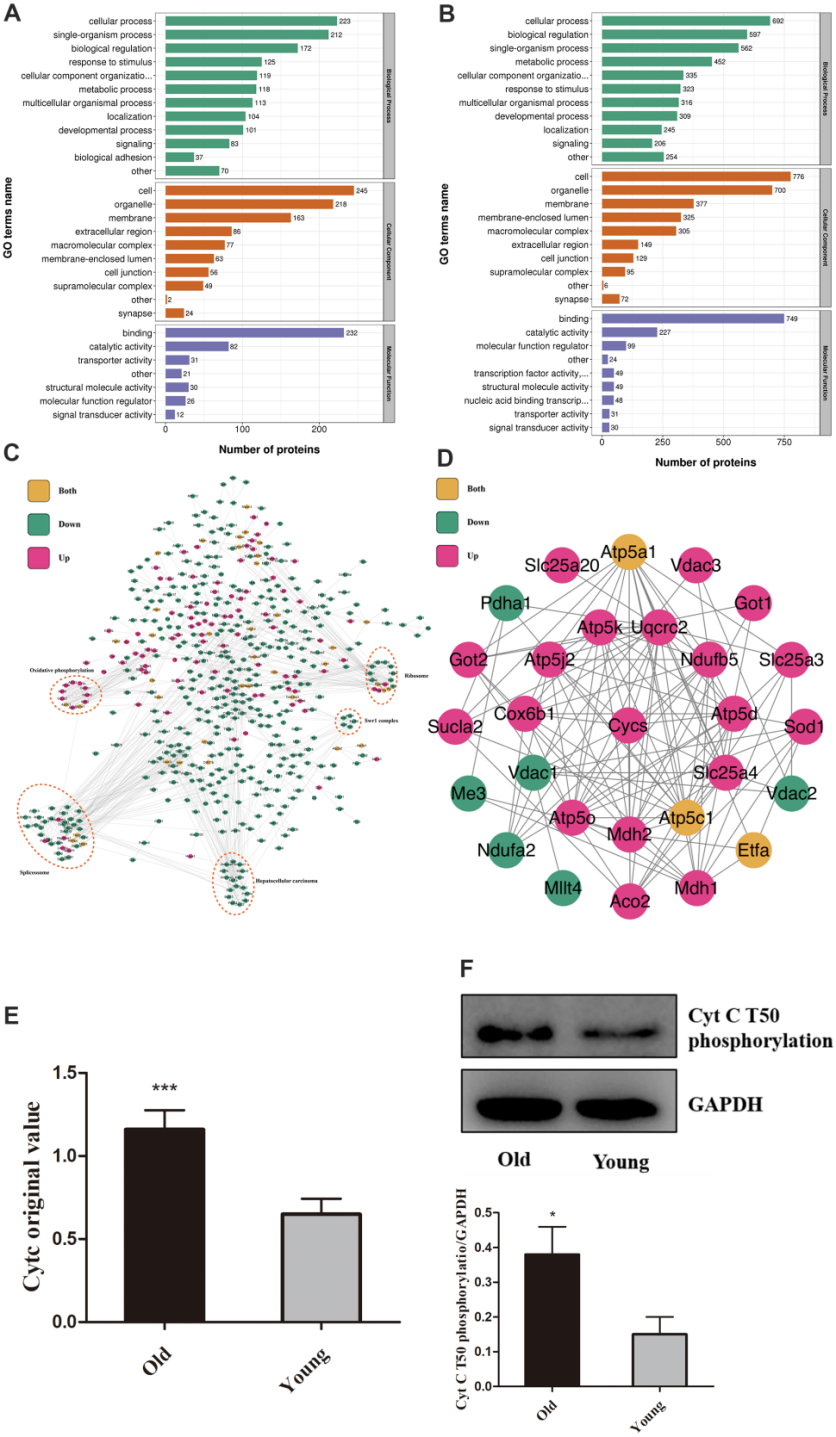
**Aging increases Cytc T50 phosphorylation.** Compared with the young, GO annotation revealed that biological function was up-regulated (**A**) in the old group and down-regulated (**B**). (**C**) Relationships between different proteins. (**D**) Magnified (Figure 1C) portion. (**E**) T50 expression is different in young and old heart tissue from proteomics. (**F**) T50 levels in isolated cardiomyocytes (from 8 weeks and 18 months-old mouse hearts) were measured by Western Blot, with specific antibody in Figure 2. n=5 in each group. Data expressed as mean±SD. *** *P* < .001 vs. young, * *P* < .05 vs. young.

**Figure 2:**
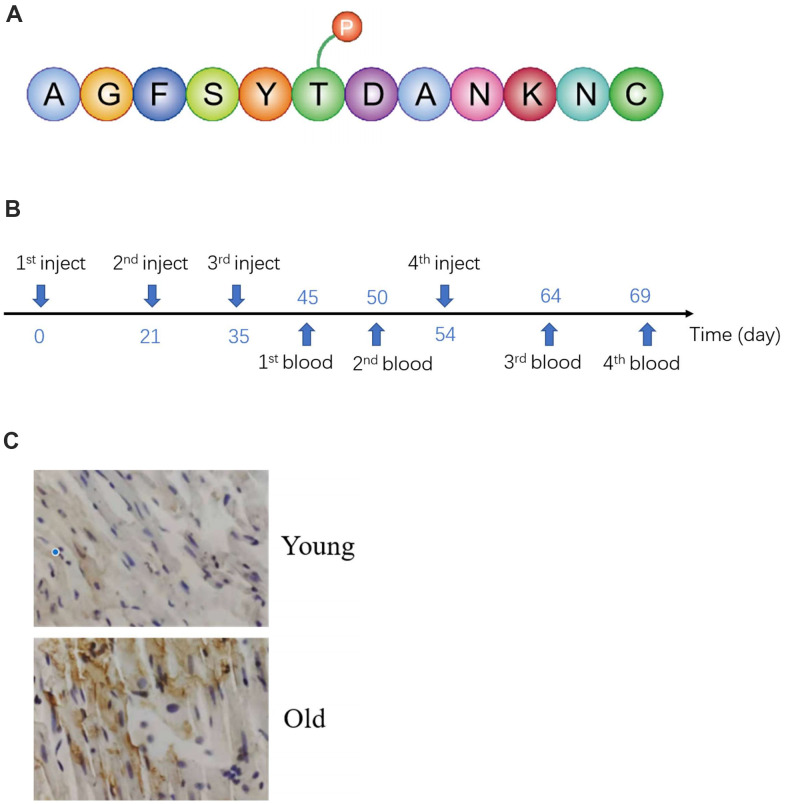
**Preparation and validation of specific antibodies.** (**A**) Antigenic peptide sequences. (**B**) Time of antigen injection and blood collection. (**C**) Validation antibody by IHC.

Phosphorylation is a post-translational modification widely existing *in vivo* and is closely related to apoptosis [12–14]. We, therefore, conducted proteomics in myocardial tissue from young and old mice to determine aging regulation on myocardial protein phosphorylation. A total of 9609 phosphorylation sites were identified on 2957 proteins, of which 8195 sites on 2747 proteins contained quantitative information. Using *P*-value<0.05 as a cut-off, the change in the amount of differential modification >1.3 was considered as significantly up-regulated and less than 1/1.3 as significantly down-regulated. (Figure 1A, 1B) The classification of proteins with statistical differences were based on the GO annotation. Respectively, Figure 1A, 1B showed the up-regulation and down-regulation of biological function in the old group. The differentially modified proteins were compared with STRING (V.10.5) protein network interaction database to determine the protein interaction network (Figure 1C). Phosphorylation changes have occurred in many essential apoptotic regulatory proteins, among which the T50 phosphorylation of Cytc attracted our attention. The level of this change was very significant (1.779 times greater in the old group than the young), and Cytc is closely related to some of the apoptosis regulators by bioinformatics analysis (Figure 1D). Surprisingly, this site has not been functionally annotated. Omics results showed a difference in its content between the old and young groups (*P* < .001, Figure 1E). We verified this result by Western Blot (*P* < .05, Figure 1F) by utilizing the antibody prepared in Figure 2.

### Preparation and validation of specific antibodies

To verify the omics results, specific antibodies were prepared. Using specific antigenic peptide sequences from NCBI (Figure 2A), they were injected into New Zealand White Rabbits four times (Figure 2B). Antibodies were extracted from rabbit blood. The efficacy of the antibody was verified by IHC and Western blot. As illustrated in Figure 2C, the intensity of immunostaining for T50 phosphorylation was markedly enhanced in the old group, compared with the young. The result of Western blot also demonstrated antibody specificity (Figure 1F).

### Gene manipulation successfully altered the expression of Cytc variants

**Figure 3:**
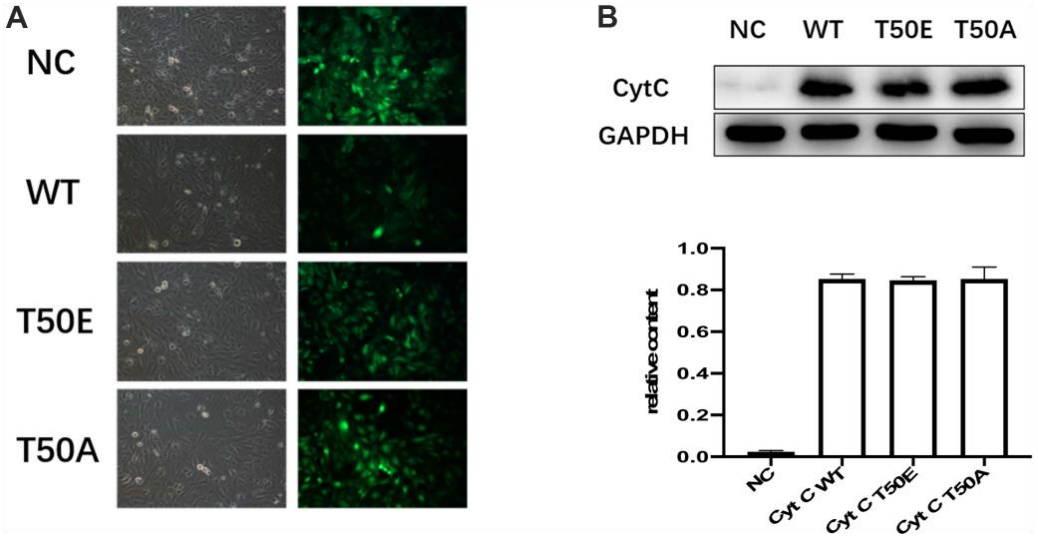
**Gene manipulation successfully altered the expression of Cytc variants.** (**A**) Transfection efficiency, 48 hours after lentiviral transfection of cardiomyocytes. Transfection efficiency is indicated by concomitant contrast and fluorescence microscopy. Lentiviral vectors carried GFP gene. Cardiomyocytes infected by Cytc variants-carrying lentivirus are identifiable by fluorescence microscopy 48 hours after infection. (**B**) Representative immunoblots of Cytc. There were no statistical differences in WT, T50E and T50A.

To study the effect of T50 phosphorylation of Cytc on apoptosis, we generated T50E phosphomimetic Cytc. Phosphomimetic amino acid replacement can functionally mimic protein phosphorylation and be used to model the functional effects of fully phosphorylated proteins [15, 16]. We also constructed human Cytc expression plasmids for WT and T50A as a non-phosphorylatable control. An empty plasmid served as a negative control. Transfection efficacy is reported in Figure 3A. No significant efficacy differences existed among the lentivirus-treated four groups. Successful expression was confirmed by Western blotting. No difference was found among the three groups (Figure 3B).

### Cytc T50 phosphorylation suppresses H/R-induced apoptosis

**Figure 4:**
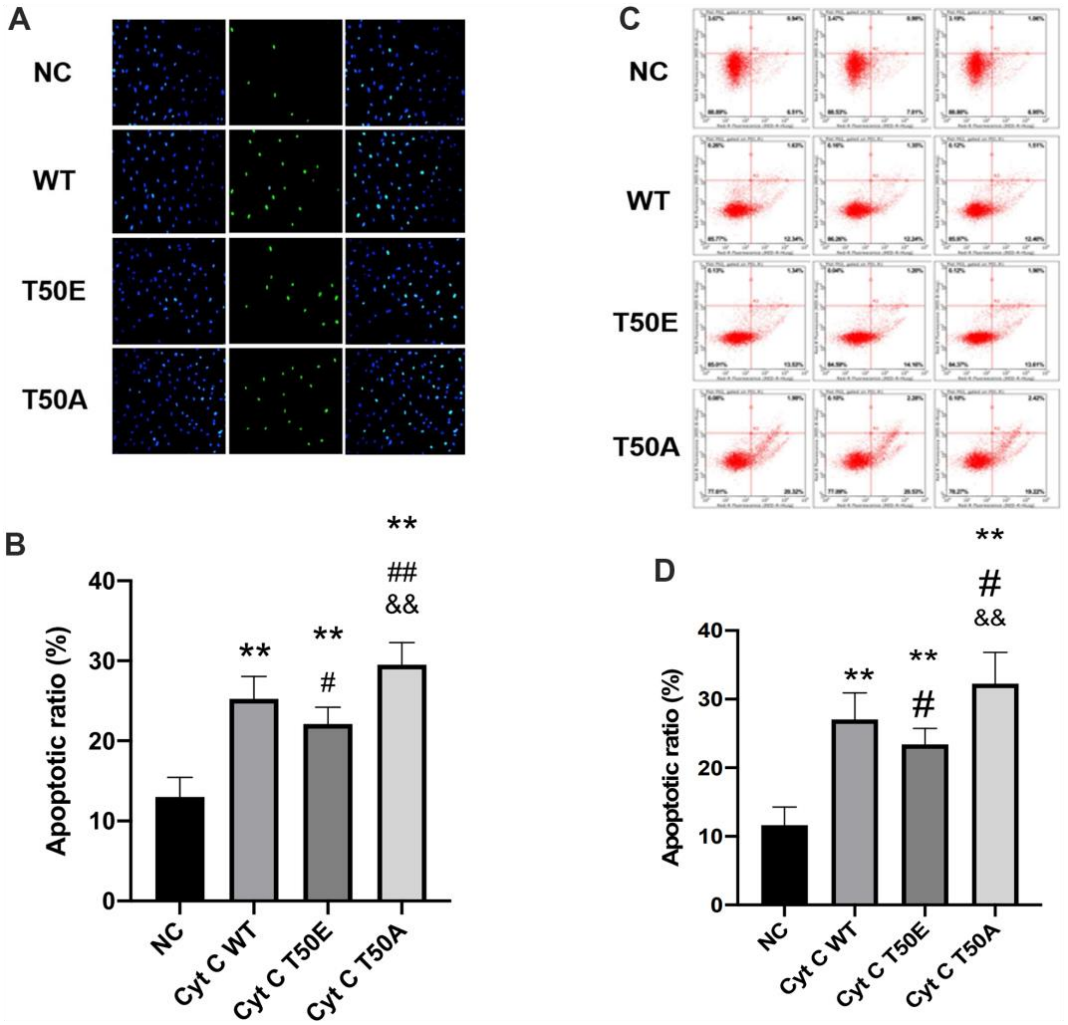
**Cytc T50 phosphorylation suppresses H/R induced apoptosis.** (**A**) Representative photomicrographs of *in situ* detection of cardiomyocytes DNA fragments from AC16 cells subjected to H/R. Cardiomyocytes were stained with DAPI (blue) and TUNEL (green). (**B**) TUNEL-positive nuclei were summarized in a graph and expressed as a percentage of all cardiomyocytes subjected to H/R. (**C**) Apoptosis in AC 16 cells were analyzed by flow cytometry. (**D**) Quantitation of apoptosis data in flow cytometry. n=3 in each group. Data expressed as mean±SD. ** *P* < .01 vs. NC, # *P* < .05 vs. WT, ## *P* < .01 vs. WT, && *P* < .01 vs. T50E.

Four types of Cytc lentiviral vectors were utilized to infect AC16 cells. After H/R treatment, the effects of gene manipulation upon cardiomyocytes apoptosis were assessed by TUNEL staining and flow cytometry analysis. Compared with NC group, TUNEL staining results showed a significant augment of total TUNEL positive nuclei in the other three groups (*P* < .01, Figure 4A, 4B). Compared with WT, the cell apoptosis rate in the T50E group was significantly lower (*P* < .05, Figure 4A, 4B). In contrast, the cell apoptosis rate is significantly higher in T50A group (*P* < .01, Figure 4A, 4B). Consequently, the cell apoptosis ratio in the T50A group was dramatically higher than T50E (*P* < .01, Figure 4A, 4B). Consistently, the results of flow cytometry analysis showed reduced apoptosis rate in T50E group (*P* < .05) and increased apoptosis in T50A group when compared with WT group (*P* < .05, Figure 4C, 4D). The ratio of apoptosis in T50A was significantly higher than T50E (*P* < .01, Figure 4C, 4D).

### Effects of T-50 phosphorylation of Cytc on caspase and Bcl-2 family activity

**Figure 5:**
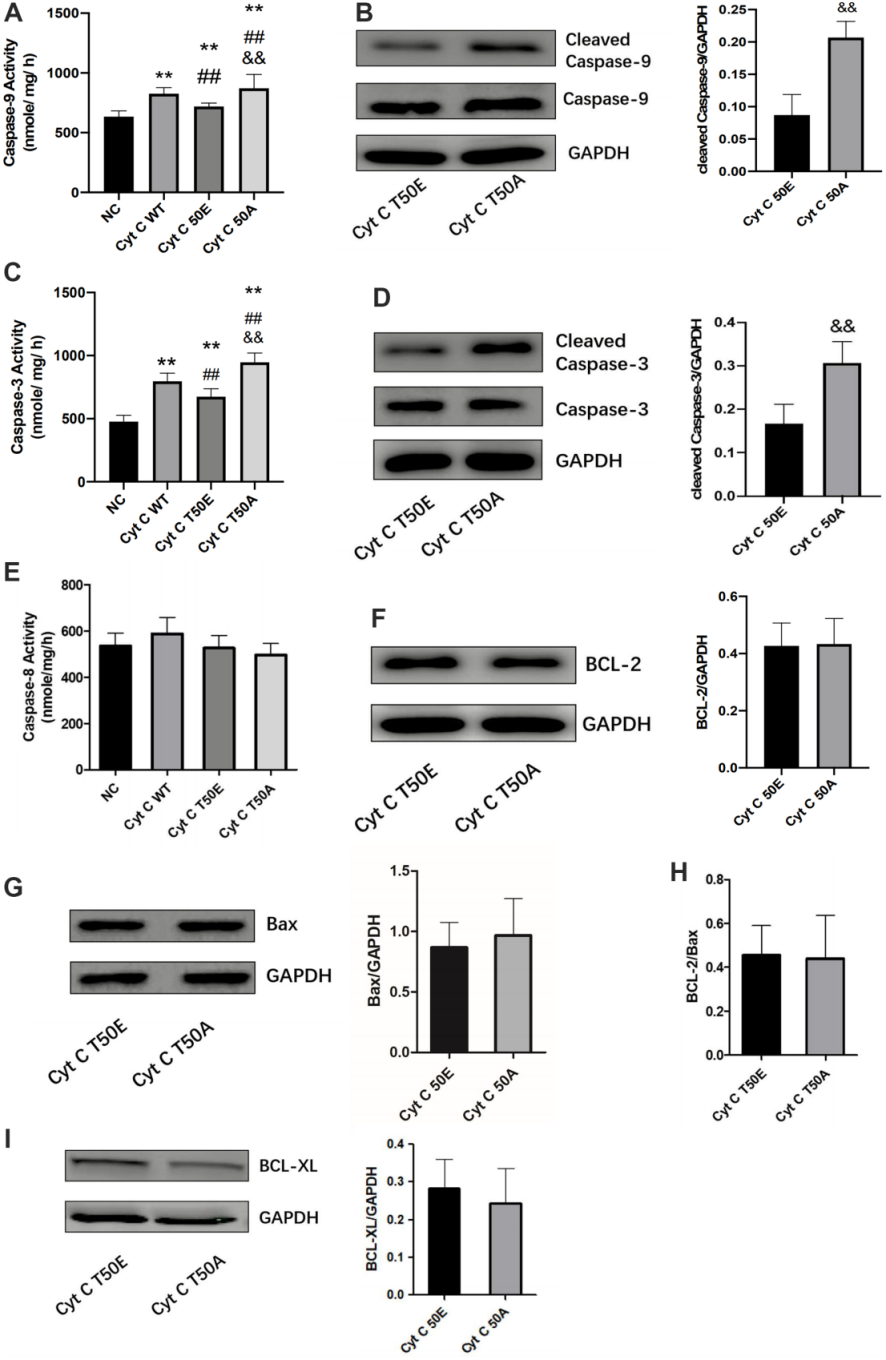
**Effects of Cytc T-50 phosphorylation on caspase and Bcl-2 family activity.** ELISA determination of caspase-9 (**A**), caspase-3 (**C**) and caspase-8 activity (**E**) in cardiomyocytes transfected with lentivirus. Representative Western blot and quantitative levels showing the expression of cleaved caspase-9 (**B**), cleaved caspase-3 (**D**), BCL-2 (**F**), Bax (**G**), BCL-2/Bax (**H**) and BCL-XL (**I**). n=3 in each group. ** *P* < .01 vs. NC, ## *P* < .01 vs. WT, && *P* < .01 vs. T50E.

Bcl-2 family members are important apoptosis regulatory factors. They can increase mitochondrial membrane permeability and promote Cytc to release from the mitochondria into the cytosol. Once Cytc is released into the cytosol, it interacts with the apoptotic protease activating factor 1 to activate the caspase-9 and the downstream caspase cascade, leading to cell death [17]. This is the general process in which Cytc participates in apoptosis. In order to study the potential mechanism of T50 phosphorylation affecting apoptosis, we detected its upstream and downstream factors. Compared with NC, caspase-9 and caspase-3 activity were significantly increased in the other three groups by ELASA assay (*P* < .01, Figure 5A, 5C). Compared with WT group, the activity of caspase-9 and caspase-3 was significantly lower in T50E group while much higher in T50A (*P* < .01, Figure 5A, 5C). T50A had a significantly higher activity of caspase-9 and caspase-3 compared with T50E (*P* < .01, Figure 5A, 5C). Western blot analysis demonstrated that cleaved caspase-9/GAPDH and cleaved caspase-3/GAPDH in T50A are higher than those in T50E (*P* < .01, Figure 5B, 5D). However, no significant difference was observed on caspase-8, BCL-2, Bax, BCL-2/Bax and BCL-XL (Figure 5E–5I).

## DISCUSSION

Previous age-related apoptosis studies, including our previous study, demonstrated that aging augments postischemia apoptosis. In 2012, through a combination of small-scale clinical trials and animal experiments, we demonstrated that aging may promote cardiac apoptosis, a significant cause of ischemia-induced heart failure [4]. Peitan L et al.’s study found that Bax mRNA / Bcl-2 mRNA and apoptosis rate are higher in aged mice compared to the young with the establishment of coronary artery ischemia/reperfusion model [18]. Data from several studies also proved this conclusion [5, 6, 19]. However, in recent years, with the in-depth research on the effects of aging on apoptosis, it has been found that aging also activates some anti-apoptotic factors. We first observed this interesting phenomenon in the mouse myocardial tissue of different ages by genomics technology [9]. Immediately thereafter, we identified that this phenomenon may also exist in humans [10]. Hence, we believe that aging promotes apoptosis, meanwhile also activating some anti-apoptotic factors.

Several novel observations were made in the present study. First, our study demonstrated that aging increases Cytc phosphorylation at T50, which has not been functionally annotated. We found it by proteomics and verified the result by generating a specific antibody. Second, phosphorylation at T50 may reduce downstream caspase family members activity to produce anti-apoptosis effects.

In this study, we extracted myocardial tissue from young and old mice and performed a proteomic experiment. The result of omics revealed extensive different phosphorylation between young and old mouse myocardial tissue. Tetsuo Y et al.’s study demonstrates that the levels of αB-crystallin phosphorylation at S59 and, to some extent, at S45 are increased in aged muscles. Phosphorylated αB-crystallin affects functional and structural properties of cardiomyocytes that are crucial for contractile function and myofibrillar organization [20]. By building phosphomimetic knock-in mouse models, Aryal N et al. proved that constitutive Dicer1 phosphorylation accelerates metabolism and aging *in vivo* [21]. Another study demonstrated that phosphorylated forms of sHsps, p38 MAPK, MK2, and ERK1/2 are elevated in aged patients’ muscles [22]. These studies have demonstrated that signaling protein phosphorylation plays an important role in aging. This conclusion is consistent with our results. In the present study, we found Cytc T50 phosphorylation by proteomics and identified that aging increases T50-phosphorylated Cytc by Western blot and IHC.

In addition to respiration, Cytc (especially its phosphorylation) is also closely related to apoptosis. Lee et al. isolated Cytc from cow heart and identified the phosphorylation site by immobilized metal affinity chromatography/nano-liquid chromatography/electrospray ionization mass spectrometry. Tyr-97 is the first phosphorylation site discovered on Cytc [23]. The other study reported that Cytc purified from the mammalian brain is phosphorylated on S47. With a recombinant phosphomimetic mutant, they found S47-phosphorylated Cytc showed lower caspase-3 activity. Phosphomimetic Cytc decreased cardiolipin peroxidase activity and is more stable in the presence of H2O2 [24]. These studies supported our results. In this study, we proved that phosphorylation Cytc at T50 has protective effects by TUNEL staining and flow cytometry analysis. Phosphorylation at this site may alter cytochrome C activity and reduce downstream caspase family members activity to produce protective effects.

In conclusion, there are several innovations in this study. First, we observed that aging increases phosphorylation Cytc at T50. Second, this aging-increasing phosphorylation site can suppress H/R-induced apoptosis. In addition, this anti-apoptotic effect is mediated mainly through inhibition of downstream caspase-9 and caspase-3 activity. Based on our previous studies [9, 10], T50-phosphorylated Cytc becomes a new anti-apoptosis factor in aging heart.

## CONCLUSIONS

In summary, our study proved a new phosphorylation site that is increased in the process of aging. More importantly, phosphorylation at this site can decrease the apoptosis ratio induced by H/R.

## MATERIALS AND METHODS

### Mouse heart harvest

Approved by the institutional ethics committee, this study was in compliance with the United States National Institutes of Health guidelines. Male C57 mice (aged eight weeks and 18 months) were anesthetized with sodium pentobarbital. Mouse hearts were harvested by exteriorizing the heart via a left thoracic incision.

### Phosphoproteomic analysis

Heart tissue from young and old mouse were harvested and delivered to PTM-Biolab Co., Ltd. (Hangzhou, China) for phosphoproteomic analysis. The quantitative study of phosphorylated proteomics used TMT labeling and phosphorylated enrichment techniques and high-resolution liquid chromatography-mass spectrometry.

### GO annotation

Gene Ontology (GO) annotation proteome was derived from the UniProt-GOA database (http://www.ebi.ac.uk/GOA/). Firstly, converting identified protein ID to UniProt ID and then mapping to GO IDs by protein ID. If some identified proteins were not annotated by UniProt GOA database, the InterProScan soft was used to annotate protein’s GO function based on the protein sequence alignment method. Then proteins were classified by Gene Ontology annotation based on three categories: biological process, cellular component, and molecular function.

### Protein-protein interaction network

All differentially expressed and/or modified protein database accession or sequence were searched against the STRING database version 10.5 for protein-protein interactions. Only interactions between the proteins belonging to the searched data set were selected, thereby excluding external candidates. STRING defines a metric called “confidence score” to define interaction confidence; we fetched all interactions that had a confidence score >0.7 (high confidence).

### Functional identification

### Antibody production

A special antibody was ordered from PTM-Biolab Co., Ltd. an antigen. Specific antibody response was examined with immunohistochemistry staining.

### Western blot

Myocardial tissue and AC16 cells were homogenized in an ice-cold lysis buffer. After homogenization, the lysates were centrifuged. The supernatant was saved and separated by electrophoresis on SDS-PAGE and transferred onto polyvinylidene difluoride-plus membranes. After blocking buffer, the immunoblots were probed with anti-T50 phosphorylation of Cytc(CL090701, PTM Bio), anti-Cytc(#ab13575, Abcam), anti-Cleaved Caspase-3(#ab184787, Abcam), anti-Caspase-3(sc-7272, Santa Cruz Biotechnology), anti-Cleaved Caspase-9(#ab2324, Abcam), anti-Caspase-9(sc-56076, Santa Cruz Biotechnology), anti-BCL-2(sc-7382, Santa Cruz Biotechnology), anti-BCL-XL(sc-8392, Santa Cruz Biotechnology), anti-Bax(sc-7480, Santa Cruz Biotechnology), and anti-GAPDH(60004-1-lg, Proteintech) antibodies overnight at 4° C, followed by incubation with fluorescent-conjugated secondary antibodies(A0208, Beyotime) at room temperature for 1 hour.

### Immunohistochemistry staining

Paraffin sections made from young and aged mouse hearts were used for immunohistochemistry assays to detect protein expression levels of T50 phosphorylation of Cytc proteins. In accordance with the manufacturer’s instruction, tissue sections stained immunohistochemically are determined separately by two pathologists using the indirect streptavidin peroxidase method. The primary antibodies against T50 (CL090701, PTM Bio) and horseradish peroxidase-conjugated IgG were used in this study. Then, the proteins were visualized *in situ* by the use of 3, 3 diaminobenzidine kit (BioGenex, Fremont, CA, USA).

### Lentivirus vector construction

Three sequences of WT, T50E and T50A were synthesized after codon optimization, purchased from Sangon Biotech Co., Ltd. (Shanghai, China). To study the effect of T50 phosphorylation *in vitro*, we generated T50E for constant phosphorylation and T50A for non-phosphorylation. Three sequences of WT, T50E and T50A are as below. *WT:*ATGGGTGATGTTGAGAAAGGCAAGAAGATTTTTATTATGAAGTGTTCCCAGTGCCACACCGTTGAAAAGGGAGGCAAGCACAAGACTGGGCCAAATCTCCATGGTCTCTTTGGGCGGAAGACAGGTCAGGCCCCTGGATACTCTTACACAGCCGCCAATAAGAACAAAGGCATCATCTGGGGAGAGGATACACTGATGGAGTATTTGGAGAATCCCAAGAAGTACATCCCTGGAACAAAAATGATCTTTGTCGGCATTAAGAAGAAGGAAGAAAGGGCAGACTTAATAGCTTATCTCAAAAAAGCTACTAATGAGTAA. *T50E:*ATGGGTGATGTTGAGAAAGGCAAGAAGATTTTTATTATGAAGTGTTCCCAGTGCCACACCGTTGAAAAGGGAGGCAAGCACAAGACTGGGCCAAATCTCCATGGTCTCTTTGGGCGGAAGACAGGTCAGGCCCCTGGATACTCTTACGAGGCCGCCAATAAGAACAAAGGCATCATCTGGGGAGAGGATACACTGATGGAGTATTTGGAGAATCCCAAGAAGTACATCCCTGGAACAAAAATGATCTTTGTCGGCATTAAGAAGAAGGAAGAAAGGGCAGACTTAATAGCTTATCTCAAAAAAGCTACTAATGAGTAA. *T5OA:*ATGGGTGATGTTGAGAAAGGCAAGAAGATTTTTATTATGAAGTGTTCCCAGTGCCACACCGTTGAAAAGGGAGGCAAGCACAAGACTGGGCCAAATCTCCATGGTCTCTTTGGGCGGAAGACAGGTCAGGCCCCTGGATACTCTTACGCCGCCGCCAATAAGAACAAAGGCATCATCTGGGGAGAGGATACACTGATGGAGTATTTGGAGAATCCCAAGAAGTACATCCCTGGAACAAAAATGATCTTTGTCGGCATTAAGAAGAAGGAAGAAAGGGCAGACTTAATAGCTTATCTCAAAAAAGCTACTAATGAGTAA. With these sequences, construct lentivirus vectors, purchased from Shanghai Yibeirui Bio-pharmaceutical Technology Co., Ltd. (Shanghai, China).

### Cell culture and transfection

Human adult ventricular cardiomyocyte cell line AC16 was purchased from the American Type Culture Collection (ATCC; Manassas, VA, USA). Cells were cultured in Dulbecco’s modified Eagle’s medium (DMEM; Invitrogen, Carlsbad, CA, USA) containing 10% fetal bovine serum (FBS; HyClone, Logan, UT, USA) and 1% penicillin/streptomycin in a humidified incubator with 5% CO2 at 37° C. In this study, four types of lentiviruses were used to infect AC16 cells. Infection efficiency was monitored by fluorescence microscopy 48 hours post lentivirus infection.

### H/R-induced cardiomyocyte apoptosis

***In vitro cardiomyocyte H/R model***

Seventy-two hours after cardiomyocyte transfection, all groups were treated with hypoxia (95% N2 and 5% CO2) for 6 hours (via Anaerobic Bag, Becton Dickinson and Company Inc., Franklin Lakes, NJ, USA), followed by normoxia (74% N2, 21% O2, and 5% CO2) for 2 hours.

### TUNEL staining

Cardiomyocytes were cultured directly upon coverslips. TUNEL staining was performed per the manufacturer’s instructions (In Situ Cell Death Protection Kit, Fluorescein, Roche, Indianapolis, IN, USA). Total nuclei were stained by DAPI (Vector Laboratories Inc., Burlingame, CA, USA). Apoptotic index (i.e., the number of positively stained nuclei/total number of nuclei counted × 100%) was determined via blinded manner.

### Flow cytometry analysis

After incubation for two days, the harvested cells were used for apoptotic determination by two methods. First, the apoptotic rate of transfected cells was evaluated by using Annexin V APC/PI apoptosis detection kit (KeyGEN) by following the manufacturer’s instruction and then analyzed using FACScan. All tests are carried out in triplicate. Second, by following the manufacturer’s instructions, Caspase-9 and Caspase-3 levels were detected utilizing human Caspase-9 ELISA kit (BC3890), Caspase-3 ELISA kit (BC3830) and Caspase-8 ELISA kit (BC3880) from Beijing Solarbio Science and Technology Co., Ltd. (Beijing, China).

### Statistical analysis

The data were analyzed with Prism 8.0 (GraphPad Software, San Diego, CA, USA). All values in the text and figures are presented as mean ± SD. Statistical differences were determined by Student’s t-test for comparison between 2 groups and ANOVA followed by Bonferroni multiple comparison test for comparison among ≥3 groups. Probabilities of.05 or less were considered statistically significant.

### Data availability

The data used to support the findings of this study are available from the corresponding author upon request.

### Limitation

In this study, phosphoproteomics were performed only in myocardial tissue from young and old mice, which reduces the targeting of the experiment. The relationship between T50 phosphorylation and mitochondrial release or caspase 9 interaction will be explored in the next step. In addition, the functional impact of Cytc T50 phosphorylation *in vivo* or clinical significance should be explored in the future.
